# The Leukocyte Subtype Counts and Ratios Can Effectively Predict the Risk of Arterial Stiffness Assessed by Cardio-Ankle Vascular Index: A Retrospective Study

**DOI:** 10.3389/fcvm.2021.671885

**Published:** 2021-05-26

**Authors:** Yaoling Wang, Ruiyun Wang, Lijuan Bai, Yun Liu, Lihua Liu, Linfeng He, Benling Qi

**Affiliations:** Department of Geriatrics, Union Hospital of Tongji Medical College, Huazhong University of Science and Technology, Wuhan, China

**Keywords:** arterial stiffness, atherosclerosis, cardio-ankle vascular index, monocyte, lymphocyte, monocyte to lymphocyte ratio

## Abstract

**Background:** Arterial stiffness was the pathological basis and risk factor of cardiovascular diseases, with chronic inflammation as the core characteristic. We aimed to analyze the association between the arterial stiffness measured by cardio-ankle vascular index (CAVI) and indicators reflecting the inflammation degree, such as count of leukocyte subtypes, platelet, and monocyte-to-lymphocyte ratio (MLR), etc.

**Methods:** The data of inpatients from November 2018 to November 2019 and from December 2019 to September 2020 were continuously collected as the training set (1,089 cases) and the validation set (700 cases), respectively. A retrospective analysis of gender subgroups was performed in the training set. The association between inflammatory indicators and CAVI or arterial stiffness by simple linear regression, multiple linear regression, and logistic regression was analyzed. The effectiveness of the inflammation indicators and the CAVI decision models to identify arterial stiffness by receiver operating curve (ROC) in the training and validation set was evaluated.

**Results:** The effect weights of MLR affecting the CAVI were 12.87% in men. MLR was the highest risk factor for arterial stiffness, with the odds ratio (95% confidence interval) of 8.95 (5.04–184.79) in men after adjusting the covariates. A cutpoint MLR of 0.19 had 70% accuracy for identifying arterial stiffness in all participants. The areas under the ROC curve of the CAVI decision models for arterial stiffness were >0.80 in the training set and validation set.

**Conclusions:** The MLR might be a high-risk factor for arterial stiffness and could be considered as a potential indicator to predict arterial stiffness.

## Introduction

Arterial stiffness is characterized by degradation in the extracellular matrix of the mediator layer, while atherosclerosis is caused by the accumulation of lipids in the intimal layer initiating the migration of inflammatory cells (1). Arterial stiffness and atherosclerosis are both processes of progressive destruction of blood vessel walls, which are an important part of the vascular aging process (2) and an independent predictor of cardiovascular or cerebrovascular events and mortality (3, 4). They share risk factors of age, hypertension (HTN), and insulin resistance; share the pathological features of impaired endothelial cell function, down-regulation of Nitric Oxide (NO) activity, and content; and both involve the pathological state of chronic inflammation (1). In particular, inflammatory cell transformation plays a key role in the pathogenesis of atherosclerosis: monocytes transform into tissue macrophages and finally produce foam cells, which is regarded as a sign of neonatal atherosclerosis (5). The leukocytes and their subtypes play a major role in repairing and replacing necrotic tissue, and the intensity of the inflammatory response is reflected by the count (6). In addition to the count of cells, the ratios between them, such as neutrophil-to-lymphocyte ratio (NLR) (7), monocyte-to-lymphocyte ratio (MLR) (8), and platelet-to-lymphocyte ratio (PLR) (9), are also considered to reflect the inflammation degree. Verdoia et al. (10) found that in people receiving coronary angiography, NLR was independently related to the prevalence and severity of coronary heart disease, and PLR (11) had a similar effect too; MLR could be an independent predictor of the severity of carotid artery stenosis in patients with ischemic stroke, and it can effectively reflect the severity of coronary artery disease better than NLR (12, 13), which diseases are serious adverse events of arterial stiffness. Therefore, we hypothesized that leukocyte counts, platelet counts, and their ratios as indicators of inflammation might be related to arterial stiffness. Intima media thickness (IMT) of the carotid artery (14), pulse-wave velocity (PWV), and cardio-ankle vascular index (CAVI) are all indicators reflecting the degree of arterial stiffness. Increased leukocyte subtype count is an independent risk factor for the occurrence and development of subclinical carotid arterial stiffness (15–17). Leukocyte and granulocyte counts were significantly positively correlated with PWV but have nothing to do with the counts of lymphocytes. However, PWV is affected by the blood pressure level at the time of measurement. The further developed CAVI was introduced into the stiffness parameter β (18, 19), and its accuracy in measuring arterial elasticity was not affected by fluctuations in blood pressure during the measurement. Therefore, we use CAVI as an indicator of arterial elasticity to analyze the association between leukocyte subtype counts, platelet counts, their ratios, and arterial stiffness.

## Materials and Methods

All materials have been made publicly available at the HARVARD Data verse and can be accessed at https://doi.org/10.7910/DVN/SGHUV2. This was a retrospective, cross-sectional study, and all samples do not contain personal identification. We were unable to obtain written consent from all patients. The study protocol conforms to the ethical guidelines of the 1975 Declaration of Helsinki.

We evaluated the arterial stiffness risk of the indicators and constructed CAVI decision models in the training set. The predictive capability for arterial stiffness of the multiple regression models was constructed in the training set, and these indicators were validated in the training set and the validation set.

To construct the decision model of CAVI, the data of patients who were hospitalized in the General Medical and Geriatrics Department of Wuhan Union Hospital from November 2018 to November 2019 and accepted arterial elasticity measurements were collected as a training set. And data from December 2019 to September 2020 were applied to verify the identifying validity for arterial stiffness of the indicators and the constructed models. Samples where the leukocyte subtype counts or ratio may be severely affected by diseases or medicine were excluded. The specific exclusion criteria are as follows: (1) definite or suspicious blood system diseases, such as infectious mononucleosis, leukemia, and lymphoma, etc.; (2) currently or recently used immunosuppressive agents, such as cyclophosphamide, cyclosporine, etc.; (3) suffering from autoimmune diseases that seriously affect the number of white blood cells, such as systemic lupus erythematosus and Graves disease; (4) severe hypersplenism; (5) taking medication that may affect the white blood cell count or infusing blood; (6) suffering from other malignant tumors and are undergoing radiotherapy or chemotherapy.

The participants' smoking and alcohol history and clinical diagnosis including HTN and diabetes mellitus (DM) were collected simultaneously. Ten milliliters of venous blood in the fasting state in the morning after the patients were admitted to the hospital were obtained, and the samples were sent to the laboratory immediately. Blood counts were evaluated by BC-3000 auto hematology analyzer (Mindray 50 Medical International, Inc.). Then, the NLR, MLR, and PLR as the ratio of neutrophils to lymphocytes, monocytes to lymphocytes, and platelets to lymphocytes were calculated, respectively. The levels of uric acid, triglycerides (TGs), cholesterol (CHOL), low-density lipoprotein, and fasting blood glucose were determined by Union Hospital's standard biochemical index laboratory measurement procedure. The NLR, MLR, and PLR were calculated as the ratio of neutrophil count to lymphocyte count, monocyte count to lymphocyte count, and platelet count to lymphocyte count, respectively.

### Arterial Stiffness Grouping

The CAVI adopts the pulse-wave algorithm and utilizes the stiffness parameter β to reduce the influence of blood pressure fluctuations on the detection of vascular elasticity. The arterial elasticity of the patient was measured with the VS-1000 AS tester of Japan Foton Company. The patient rested quietly for about 15 min before the examination and then measured in the supine position. The blood pressure of the extremities at the position of the upper arm and ankle arteries was measured, and the electrocardiogram, heart sound graph, and pulse-wave waveform were recorded at the same time. The bilateral CAVI (m/s) value was calculated, and the average of the measured values on both sides was employed in the study. Arterial stiffness is considered as CAVI ≥9 (m/s); otherwise, it is non-arterial stiffness (non-AS).

### Statistical Analysis

Quantitative data of non-normal distribution and normal distribution were expressed as medians with interquartile range and mean ± SD, respectively. Categorical data were expressed as amounts with percentages. The Mann–Whitney *U* test and *t* test were used to compare the difference between non-AS and AS in non-normal distribution and normal distribution quantitative data, respectively. A χ^2^ test was used to compare the difference of categorical data between the groups. Analysis of covariance (ANCOVA) is used to compare continuous variables between groups after adjusting for confounding factors. The inflammatory indicator difference was tested between AS and non-AS on the sample set after implementing confounding variables by individually pair-matched case–control analysis. The scatter plots were drawn between CAVI and each inflammation indicator, and the fitted linear lines for each inflammation indicator were calculated. The multiple regression analyses were performed using the stepwise backward method to determine the contribution size of inflammation indicators and clinical variables to arterial stiffness. The odds ratios (ORs) and 95% confidence intervals for arterial stiffness were calculated with logistic regression after adjustment for covariates that may affect arterial elasticity. A subgroup analysis of inflammatory indicators to AS risk in people with different clinical pathophysiological characteristics was carried out. Finally, the receiver operating characteristic (ROC) curves were determined to assess discrimination of these indicators and final multiple regression models to diagnose arterial stiffness. *p* < 0.05 was considered statistically significant. All calculations and graphs were done using R (version 3.6.3-Mac OS X 10.11).

## Results

The training set and the validation set included 1,089 and 700 sample materials, respectively. The materials stratified by arterial stiffness are summarized in Table 1.

**Table 1:** Basic characteristics of the participants in training set and validation set stratified by AS.

	**Training set**					**Validation set**			
	**All**	**Non-AS**	**AS**	***P^a^***	***P^b^***	**All**	**Non-AS**	**AS**	***P^a^***
*n*	1,089	825	264			700	508	192	
Age	57.74 (14.03)	53.41 (11.42)	71.27 (12.78)	<0.001		58.89 (13.67)	54.85 (11.87)	69.57 (12.30)	<0.001
Sex, male, *n* (%)	752 (69.1)	552 (66.9)	200 (75.8)	0.009		478 (68.3)	343 (67.5)	135 (70.3)	0.537
BMI (kg/m^2^)	24.49 (3.40)	24.75 (3.35)	23.69 (3.45)	<0.001		24.23 (3.46)	24.42 (3.46)	23.75 (3.43)	0.023
CAVI (m/s)	8.17 (1.46)	7.55 (0.90)	10.10 (1.16)	<0.001		8.24 (1.51)	7.53 (0.92)	10.14 (1.05)	<0.001
Smoke, yes, *n* (%)	343 (31.50)	251 (30.40)	92 (34.8)	0.204		203 (29.00)	151 (29.7)	52 (27.1)	0.553
Alcohol, yes, *n* (%)	305 (28.00)	240 (29.10)	65 (24.6)	0.184		168 (24.00)	137 (27.0)	31 (16.1)	0.004
LY (G/L)	1.80 [1.43, 2.20]	1.84 [1.50, 2.23]	1.60 [1.26, 2.00]	<0.001	0.914	1.76 [1.40,2.41]	1.80 [1.47, 2.16]	1.52 [1.19, 2.04]	<0.001
NE (G/L)	3.19 [2.58, 4.09]	3.10 [2.51, 3.93]	3.52 [2.72, 4.41]	<0.001	<0.001	3.17 [2.56,4.00]	3.13 [2.54, 3.93]	3.23 [2.61, 4.12]	0.111
MO (G/L)	0.37 [0.27, 0.47]	0.34 [0.27, 0.45]	0.41 [0.32, 0.53]	<0.001	<0.001	0.36 [0.27,0.46]	0.35 [0.26, 0.45]	0.38 [0.29, 0.47]	0.018
PLT (G/L)	207.00 [169.00, 242.00]	209.00 [175.00, 245.00]	196.50 [154.75, 234.00]	<0.001	0.063	205.00 [173.00,239.00]	209.50 [178.00, 245.00]	191.50 [157.50, 226.50]	<0.001
NLR	1.77 [1.37, 2.42]	1.68 [1.31, 2.25]	2.09 [1.66, 3.08]	<0.001	0.003	1.83 [1.40,2.41]	1.72 [1.36, 2.27]	2.12 [1.66, 2.82]	<0.001
MLR	0.20 [0.15, 0.27]	0.19 [0.14, 0.24]	0.25 [0.19, 0.35]	<0.001	<0.001	0.21 [0.15,0.28]	0.20 [0.14, 0.26]	0.23 [0.18, 0.32]	<0.001
PLR	115.54 [90.87, 142.55]	114.44 [90.87, 137.97]	122.67 [90.54, 156.06]	0.008	0.034	117.00 [93.40,150.61]	115.39 [93.40, 148.63]	120.65 [93.56, 159.28]	0.148
UA (μmol/L)	358.70 [292.80, 429.20]	358.10 [290.40, 431.10]	363.70 [296.57, 423.00]	0.763		356.30 [286.03,422.62]	359.50 [291.12, 429.05]	345.45 [277.25, 409.77]	0.177
FBG (mmol/L)	4.97 [4.59, 5.57]	4.92 [4.56, 5.45]	5.19 [4.68, 6.21]	<0.001		5.00 [4.59,5.56]	4.95 [4.57, 5.46]	5.24 [4.70, 5.93]	<0.001
TGs (mmol/L)	1.35 [0.97, 2.08]	1.43 [0.99, 2.19]	1.15 [0.87, 1.84]	<0.001		1.30 [0.92,1.90]	1.40 [1.00, 2.01]	1.17 [0.82, 1.62]	<0.001
CHOL (mmol/L)	4.34 [3.63, 5.08]	4.48 [3.82, 5.16]	3.96 [3.26, 4.66]	<0.001		4.24 [3.57,4.94]	4.36 [3.70, 5.04]	3.88 [3.27, 4.65]	<0.001
LDL-C (mmol/L)	2.61 [1.98, 3.21]	2.71 [2.11, 3.24]	2.24 [1.68, 2.93]	<0.001		2.46 [1.95,3.13]	2.59 [2.07, 3.17]	2.19 [1.64, 2.74]	<0.001
HTN, yes, *n* (%)	619 (56.8)	414 (50.2)	205 (77.7)	<0.001		422 (60.30)	276 (54.3)	146 (76.0)	<0.001
DM, yes, *n* (%)	237 (21.8)	145 (17.6)	92 (34.8)	<0.001		220 (34.10)	129 (25.4)	91 (47.4)	<0.001

*Values are represented as mean (SD), medians [interquartile range (IQR)], or n (%)*.

a *Between the groups, the t test and Mann–Whitney U test were used to continuity data with and without normal distribution, respectively; the χ^2^ test was used for categorical data*.

b *The differences of inflammatory indicators between AS and non-AS by analysis of covariance (ANCOVA), and covariates include age, sex, TGs, BMI, CHOL, DM, and HTN*.

*AS, arterial stiffness; non-AS, non-arterial stiffness; CAVI, cardio-ankle vascular index; BMI, body mass index; LY, lymphocyte; NE, neutrophil; MO, monocyte; PLT, platelet; NLR, neutrophil–lymphocyte ratio; MLR, monocyte–lymphocyte ratio; PLR, platelet–lymphocyte ratio; UA, uric acid; FBG, fasting blood glucose; TGs, triglycerides; CHOL, cholesterol; LDL-C, low-density lipoprotein; HTN, hypertension; DM, diabetes*.

### Participants' Characteristics at Baseline Assessment in Training Set

In training set, the counts of cells and their ratios were significantly different between the two groups. The neutrophils (NE), monocytes (MO), NLR, MLR, and PLR of participants with arterial stiffness were significantly higher than those of patients without arterial stiffness, whereas lymphocytes (LY) and platelets (PLT) were lower in the AS group. Then, with age, sex, BMI, TGs, CHOL, HTN, and DM as covariates, which show a significant difference between the AS and non-AS groups, we further performed the ANCOVA to test the differences of leukocyte counts and their ratios between patients with and without AS. The results indicated that after adjusting the confounding variables, MO (*p* < 0.001), NE (*p* < 0.001), MLR (*p* < 0.001), NLR (*p* = 0.003), and PLR (*p* = 0.034) remained significant differences in the group of AS and non-AS, whereas PLT (*p* = 0.063) and LY (*p* = 0.914) were not significantly different between the groups (Table 1).

### Individually Pair-Matched Case–Control Study From Training Set

To alleviate the influence from age, sex, BMI, dyslipidemia, and the differences in the distribution of HTN and DM between AS and non-AS in Table 1, we designed an individually pair-matched case–control analysis. The data set comes from the training set. We designed matching according to the ratio of the sample size of the case group (AS) to the control group (non-AS) as 1:2. The matching elements include the baseline materials in Table 1, which have significant differences between the AS and non-AS groups: the age group, sex, BMI group, dyslipidemia group, HTN, and DM. Finally, 116 samples of the case group (AS) were matched with 232 samples of the control group (non-AS).

The material after matching confounding factors was tested for differences between groups of AS and non-AS. The results showed that the age group, sex, BMI, various types of blood lipids, and distribution of HTN and DM were no longer significantly different between the groups, demonstrating matching successfully. The difference of LY, PLT, and PLR between AS and non-AS groups disappeared, whereas NE, MO, NLR, and MLR in the AS group were still significantly higher than those in the non-AS group (Table 2).

**Table 2:** Individually pair matched case–control analysis for AS from training set.

	**All**	**Non-AS**	**AS**	***p*^b^**
**Individually pair matched case–control set^a^**				
*n*	348	232	116	
Age	62.71 (14.03)	61.51 (10.87)	65.12 (13.43)	0.007
**Age group** ***n*** **(%)^c^**				1
Young	30 (8.6)	20 (8.6)	10 (8.6)	
Middle-aged	138 (39.7)	92 (39.7)	46 (39.7)	
Elderly	180 (51.7)	120 (51.7)	60 (51.7)	
Sex, men, *n* (%)	276 (79.3)	184 (79.3)	92 (79.3)	1
BMI (kg/m^2^)	24.83 (3.25)	24.89 (3.20)	24.72 (3.37)	0.637
**BMI group**, ***n*** **(%)^d^**				1
Low weight	3 (0.9)	2 (0.9)	1 (0.9)	
Normal	144 (41.4)	96 (41.4)	48 (41.4)	
Obesity	51 (14.7)	34 (14.7)	17 (14.7)	
Over weight	150 (43.1)	100 (43.1)	50 (43.1)	
Hyperlipidemia, *n* (%)^e^	72 (20.7)	48 (20.7)	24 (20.7)	1
CAVI (m/s)	8.55 (1.48)	7.79 (0.90)	10.06 (1.23)	<0.001
Smoke, yes, *n* (%)	134 (37.9)	88 (37.9)	46 (39.7)	0.846
**Alcohol, yes**, ***n*** **(%)**	115 (35.3)	82 (35.3)	33 (28.4)	0.243
LY (G/L)	1.69 [1.36, 2.06]	1.65 [1.40, 2.05]	1.71 [1.30, 2.10]	0.883
NE (G/L)	3.20 [2.57, 4.05]	3.04 [2.51, 3.76]	3.65 [2.67, 4.70]	0.001
MO (G/L)	0.38 [0.29, 0.50]	0.37 [0.27, 0.46]	0.42 [0.33, 0.57]	<0.001
PLT (G/L)	197.00 [162.75, 231.00]	191.00 [161.00, 226.25]	204.50 [166.00, 234.00]	0.057
NLR	1.84 [1.43, 2.57]	1.77 [1.39, 2.37]	1.95 [1.62, 2.91]	0.004
MLR	0.22 [0.17, 0.30]	0.21 [0.16, 0.27]	0.25 [0.19, 0.33]	<0.001
PLR	115.92 [91.61, 139.79]	115.31 [91.12, 136.73]	120.25 [99.63, 149.22]	0.167
UA (μmol/L)	361.20 [310.175, 425.200]	357.85 [308.08, 425.20]	368.30 [320.48, 425.68]	0.342
FBG (μmol/L)	5.03 [4.62, 5.78]	5.00 [4.61, 5.60]	5.16 [4.65, 6.12]	0.133
TGs (mmol/L)	1.33 [0.96, 1.95]	1.34 [0.93, 1.96]	1.24 [1.02, 1.94]	0.839
CHOL (mmol/L)	4.08 [3.40, 4.83]	4.08 [3.38, 4.91]	4.06 [3.49, 4.79]	0.843
LDL-C (mmol/L)	2.40 [1.87, 3.06]	2.38 [1.87, 3.05]	2.41 [1.92, 3.06]	0.841
HTN, yes, *n* (%)	234 (67.2)	156 (67.2)	78 (67.2)	1
DM, yes, *n* (%)	69 (19.8)	46 (19.8)	23 (19.8)	1

*Values are represented as mean (SD), medians [interquartile range (IQR)], or n (%)*.

a *The data set derived from the training set with matching according to the ratio of the cases (AS) and controls (non-AS) as 1:2. The matching elements include sex, the age group, the BMI group, hyperlipidemia, HTN, and DM*.

b *Between the groups, the t test and Mann–Whitney U test were used to continuity data with and without normal distribution, respectively; the χ^2^ test was used for categorical data*.

c *The age group, all training samples are divided into three equal parts according to age: young (age <51 years), middle-aged (51 ≤ age <62 years) and elderly (age ≥62 years)*.

d *The BMI group, all training samples are divided into low weight (BMI ≤ 18.5 kg/m^2^), the normal (18.5 kg/m^2^ < BMI ≤ 23.9 kg/m^2^), overweight (23.9 kg/m^2^ < BMI ≤ 27.9 kg/m^2^), and obesity (BMI > 27.9 kg/m^2^)*.

e *Hyperlipidemia (20) (TGs ≥ 2.3 mmol/L or CHOL ≥ 6.2 mmol/L or LDL-C ≥ 4.1 mmol/L) and the normal (TGs <2.3 mmol/L and CHOL <6.2 mmol/L and LDL-C <4.1 mmol/L)*.

### Risk Assessment and Model Construction in Training Set

**Figure 1:**
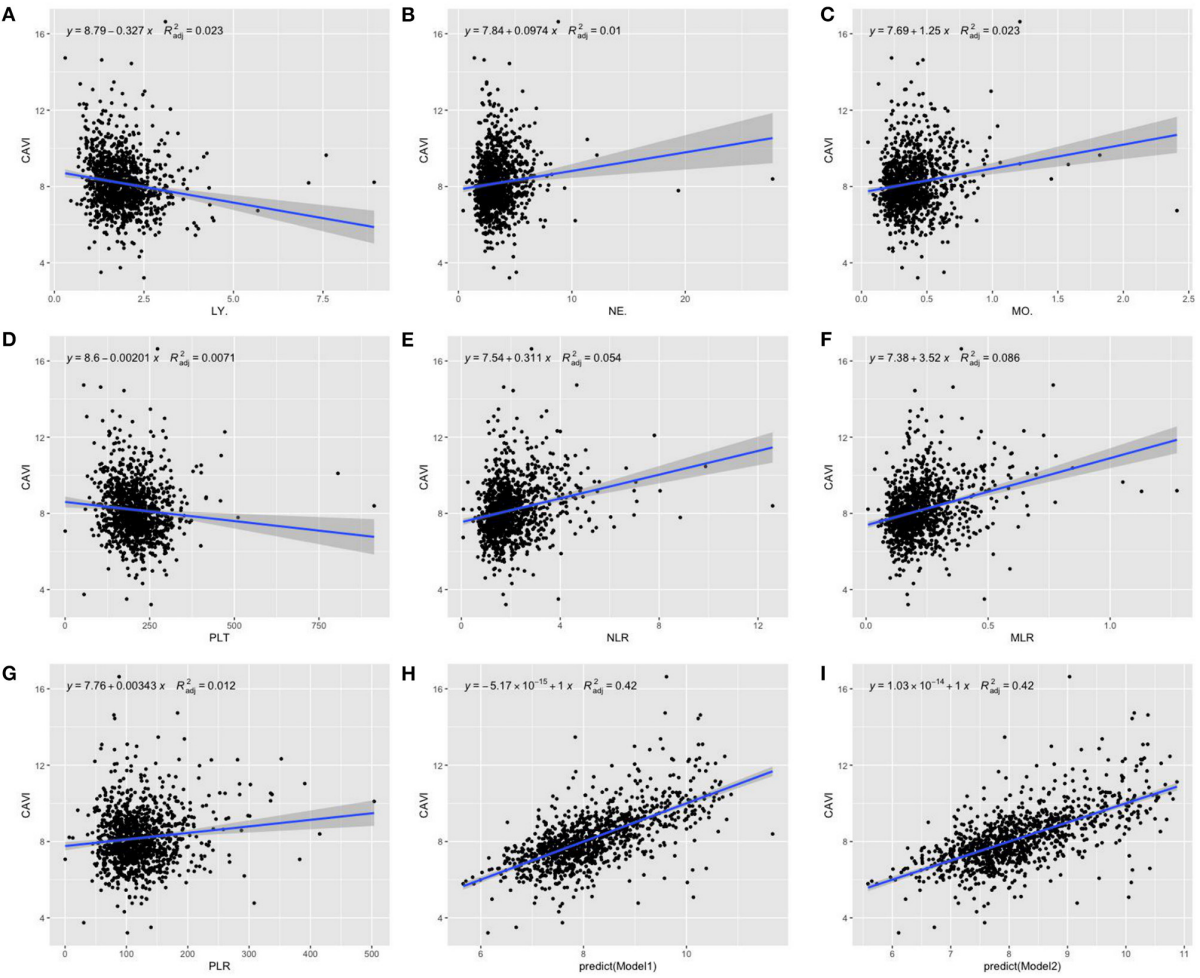
Scatterplot diagrams and linear fitting curves of LY counts **(A)**, NE counts **(B)**, MO counts **(C)**, PLT counts **(D)**, NLR **(E)**, MLR **(F)**, PLR **(G)**, predicted values of model 1 **(H)**, and model 2 **(I)** vs. CAVI in total population. CAVI, cardio-ankle vascular index (m/s); LY, lymphocyte (G/L); NE, neutrophil (G/L); MO, monocyte (G/L); PLT, platelet (G/L); NLR, neutrophil–lymphocyte ratio; MLR, monocyte–lymphocyte ratio; PLR, platelet–lymphocyte ratio; BMI, body mass index; HTN, hypertension; DM, diabetes; *R*^2^adj, adjusted *R*^2^. Model 1: The model of multivariate linear regression analysis with backward regression method of leukocyte subtype counts, platelet counts, and clinical data. The final independent variables were composed of age, sex, BMI, HTN, DM, NE, and MO. Model 2: The model of multivariate linear regression analysis with backward regression method of NLR, MLR, PLR, and clinical data. The final independent variables were composed of age, sex, BMI, HTN, DM, and MLR.

**Figure 2:**
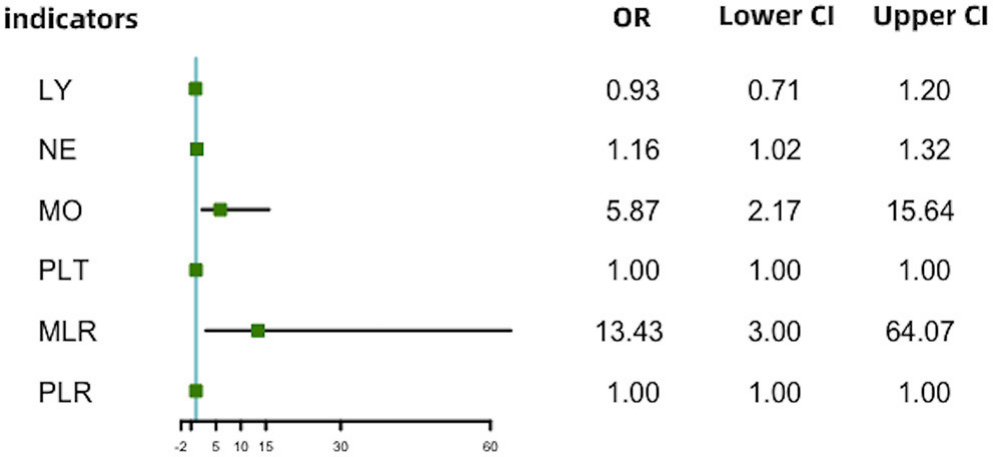
Forest plot of inflammatory indicators for arterial stiffness risk in all participants of training set. LY, lymphocyte (G/L); NE, neutrophil (G/L); MO, monocyte (G/L); PLT, platelet (G/L); NLR, neutrophil–lymphocyte ratio; MLR, monocyte–lymphocyte ratio; PLR, platelet–lymphocyte ratio.

With scatter diagrams and linear regression method, we demonstrated the association between CAVI and leukocyte subtype counts, CAVI and platelet counts, and CAVI and their ratios, respectively (Figure 1). The results of univariate linear regression are summarized in Table 3. Multivariate linear regression analysis with backward regression method was performed to determine the independent predictors of CAVI. Because of the strong collinearity [Variance Inflation Factor (VIF) >5] between leukocyte subtype counts and their ratios that were simultaneously incorporated into the multiple linear regression equation, we involved them into the two models in the same population. Models 1, 3, and 5 contain counts of cells and general clinical data in total population, in men and women, respectively. Models 2, 4, and 6 consist of NLR, MLR, PLR, and general clinical data in total population, in men and women, respectively. The final model parameters are summarized in Table 4. In the total population and men, MLR had a relatively major impact on CAVI (models 2, 4), and its weights on CAVI in the two models were 9.88 and 12.87%. The forest plot of inflammatory indicators for arterial stiffness risk in all participants of training set is shown in Figure 2. The OR of MO is significantly higher than those of NE, LY, and PLT, which were 6.95–12.38 in different populations. The ORs of MLR for arterial stiffness were 494.21 (137.74–1,884.73) in the total population, 897.37 (192.70–4,562.99) in men, and 71.69 (7.47–827.64) in women, which were much higher than all other indicators. After adjusting the baseline characteristics, that risk had been reduced and was no longer significant in the women (Table 5).

**Table 3:** The univariate linear regression analysis for the inflammatory indicators in all participants, in men and women.

	**Regression coefficients**	**95% CI**	**Adjusted *R*^**2**^**	***p***
LY^a^	−0.332	−0.453 to −0.211	0.024	<0.001
NE^a^	0.102	0.049 to 0.156	0.012	<0.001
MO^a^	1.248	0.781 to 1.714	0.023	<0.001
PLT^a^	−0.002	−0.003 to 0.084	0.007	0.003
NLR^a^	0.311	0.236 to 0.387	0.054	<0.001
MLR^a^	3.521	2.851 to 4.191	0.086	<0.001
PLR^a^	0.003	0.002 to 0.005	0.012	<0.001
LY^b^	−0.487	−0.633 to −0.340	0.052	<0.001
NE^b^	0.100	0.021 to 0.179	0.007	0.014
MO^b^	1.150	0.516 to 1.784	0.015	<0.001
PLT^b^	−0.003	−0.005 to −0.001	0.011	0.003
NLR^b^	0.138	0.088 to 0.189	0.036	<0.001
MLR^b^	3.761	2.981 to 4.541	0.106	<0.001
PLR^b^	0.005	0.003 to 0.007	0.031	<0.001
LY^c^	0.043	−0.176 to 0.262	−0.003	0.699
NE^c^	0.126	0.005 to 0.205	0.025	0.002
MO^c^	1.289	0.573 to 2.005	0.033	<0.001
PLT^c^	3.385e−05	−0.002 to 0.002	−0.003	0.975
NLR^c^	0.165	0.037 to 0.294	0.016	0.012
MLR^c^	2.232	0.942 to 3.522	0.031	<0.001
PLR^c^	0.001	0.003 to 0.007	−0.002	0.680

a *In all participants*.

b *In men*.

c *In women*.

*CAVI, cardio-ankle vascular index; LY, lymphocyte; NE, neutrophil; MO, monocyte; NLR, neutrophil–lymphocyte ratio; MLR, monocyte–lymphocyte ratio; PLR, platelet–lymphocyte ratio*.

**Table 4:** Multiple linear regression analysis of CAVI in all participants, in men and women.

	**Model 1**			**Model 2**			**Model 3**			**Model 4**			**Model 5**			**Model 6**		
	**β**	***p***	**Weights**	**β**	***p***	**Weights**	**β**	***p***	**Weights**	**β**	***p***	**Weights**	**β**	***p***	**Weights**	**β**	***p***	**Weights**
Age	0.06	<0.001	77.36	0.06	<0.001	72.37	0.06	<0.001	84.78	0.06	<0.001	76.18	0.05	<0.001	65.96	0.05	<0.001	63.98
Sex	−0.31	<0.001	2.23	−0.31	<0.001	2.19	-	-	-	-	-	-	-	-	-	-	-	-
BMI	−0.07	<0.001	6.36	−0.06	<0.001	5.87	−0.06	<0.001	9.50	−0.05	<0.001	8.68	−0.07	<0.001	3.50	−0.08	<0.001	4.29
HTN	0.19	0.016	6.55	0.20	0.009	6.56	-	-	-	-	-	-	0.453	0.001	18.85	0.14	0.001	18.72
DM	0.17	0.047	3.03	0.19	0.026	3.13	0.18	0.063	2.23	0.202	0.035	2.27	-	-	-	-	-	-
LY	-	-	-	-	-	-	-	-	-	-	-	-	0.17	0.089	0.92	-	-	-
NE	0.06	0.025	1.80	-	-	-	0.08	0.025	1.40	-	-	-	0.06	0.032	3.95	-	-	-
MO	0.40	0.218	2.68	-	-	-	0.45	0.121	2.08	-	-	-	-	-	-	-	-	-
MLR	-	-	-	0.79	0.007	9.88	-	-	-	1.19	<0.001	12.87	-	-	-	-	-	-
FBG	-	-	-	-	-	-	-	-	-	-	-	-	0.11	0.007	6.81	0.04	0.011	6.50
UA	-	-	-	-	-	-	-	-	-	-	-	-	-	-	-	0.001	0.120	6.51

*Multivariate linear regression analysis with backward regression method was performed to determine the independent predictors of CAVI*.

*Model 1: In all participants; initial inclusion indicators include age, sex, BMI, HTN, DM, alcohol, smoke, LDL-C, UA, TGs, FBG, CHOL, LY, NE, MO, and PLT; R^2^adj = 0.421, AIC = 235.28*.

*Model 2: In all participants; initial inclusion indicators include age, sex, BMI, HTN, DM, alcohol, smoke, LDL-C, UA, TGs, FBG, CHOL, NLR, MLR, and PLR; R^2^adj = 0.416, AIC = 243.20*.

*Model 3: In men; initial inclusion indicators include age, BMI, HTN, DM, alcohol, smoke, LDL-C, UA, TGs, FBG, CHOL, LY, NE, MO, and PLT; R^2^adj = 0.433, AIC = 174.00*.

*Model 4: In men; initial inclusion indicators include age, BMI, HTN, DM, alcohol, smoke, LDL-C, UA, TGs, FBG, CHOL, NLR, MLR, and PLR; R^2^adj = 0.432, AIC = 174.35*.

*Model 5: In women; initial inclusion indicators include age, BMI, HTN, DM, alcohol, smoke, LDL-C, UA, TGs, FBG, CHOL, LY, NE, MO, and PLT; R^2^adj = 0.391, AIC = 52.68*.

*Model 6: In women; initial inclusion indicators include age, BMI, HTN, DM, alcohol, smoke, LDL-C, UA, TGs, FBG, CHOL, NLR, MLR, and PLR; R^2^adj = 0.383, AIC = 56.11*.

*CAVI, cardio-ankle vascular index; BMI, body mass index; HTN, hypertension; DM, diabetes; LY, lymphocyte; NE, neutrophil; MO, monocyte; MLR, monocyte–lymphocyte ratio; FBG, fasting blood glucose; UA, uric acid; R^2^adj, adjusted R^2^; AIC, Akaike information criterion*.

**Table 5:** The ORs and 95% CI of inflammatory indicators to AS by logistic regression.

	**Model 1**			**Model 2**		
	**OR**	**95% CI**	***p***	**OR**	**95% CI**	***P***
LY^a^	0.52	0.40–0.67	0.041	0.93	0.71–1.20	0.572
NE^a^	1.20	1.09–1.33	<0.001	1.16	1.02–1.32	0.025
MO^a^	11.57	5.20–26.64	<0.001	5.87	2.17–15.64	<0.001
PLT^a^	1.00	0.99–1.00	0.023	1.00	1.00–1.00	0.241
NLR^a^	1.63	1.43–1.87	<0.001	1.22	1.04–1.44	0.018
MLR^a^	494.21	137.74–1,884.73	<0.001	13.43	3.00–64.07	<0.001
PLR^a^	1.01	1.00–1.01	<0.001	1.00	1.00–1.00	0.106
LY^b^	0.39	0.28–0.53	<0.001	0.87	0.63–1.18	0.389
NE^b^	1.21	1.08–1.36	0.001	1.30	1.11–1.52	0.001
MO^b^	12.38	4.67–33.92	<0.001	12.44	3.53–47.30	<0.001
PLT^b^	1.00	0.99–1.00	0.058	1.00	1.00–1.00	0.093
NLR^b^	1.80	1.53–2.13	<0.001	1.35	1.10–1.66	0.004
MLR^b^	897.37	192.70–4,562.99	<0.001	28.95	5.04–184.79	<0.001
PLR^b^	1.01	1.01–1.01	<0.001	1.00	1.00–1.01	0.038
LY^c^	0.02	0.66–1.49	0.923	1.21	0.68–2.34	0.546
NE^c^	1.14	0.99–1.36	0.092	1.03	0.87–1.20	0.716
MO^c^	6.95	1.95–30.69	0.006	2.11	0.30–12.12	0.441
PLT^c^	1.00	0.99–1.00	0.516	1.00	1.00–1.00	0.909
NLR^c^	1.29	1.04–1.63	0.023	1.02	0.76–1.33	0.889
MLR^c^	71.69	7.47–827.64	<0.001	1.30	0.07–34.09	0.867
PLR^c^	1.00	1.00–1.01	0.631	1.00	0.99–1.00	0.759

*Model 1: Uanadjusted*.

*Model 2: Adjusted for age, sex, BMI, HTN, DM, alcohol, smoke, LDL-C, UA, TGs, FBG, and CHOL*.

a *In all participants*.

b *In men*.

c *In women*.

*OR, odds ratio; CI, confidence interval; BMI, body mass index; HTN, hypertension; DM, diabetes; LDL-C, low-density lipoprotein; UA, uric acid; TGs, triglycerides; FBG, fasting blood glucose; CHOL, cholesterol; LY, lymphocyte; NE, neutrophil; MO, monocyte; PLT, platelet; NLR, neutrophil–lymphocyte ratio; MLR, monocyte–lymphocyte ratio; PLR, platelet–lymphocyte ratio*.

To study the AS risk of leukocyte counts and their ratios in populations with different clinicopathological characteristics, we further performed four subgroup analyses of the indicators including MLR to the AS risk (in DM, in HTN, in patients with elevated TGs, and in patients with increased CHOL, respectively). The results indicated that only NLR and MLR showed the significant risk for AS in all subgroup analyses, and the risk value of MLR (OR = 54.25–3,211.41) was much higher than NLR (OR = 1.29–1.93) (Table 6).

**Table 6:** Subgroup analysis of inflammatory indicators on the risk of AS.

		**NE**		**LY**		**MO**		**PLT**		**NLR**		**PLR**		**MLR**	
	***n* (%)**	**OR**	**95% CI**	**OR**	**95% CI**	**OR**	**95% CI**	**OR**	**95% CI**	**OR**	**95% CI**	**OR**	**95% CI**	**OR**	**95% CI**
DM	237 (21.8%)	1.11^a^	0.92–1.33	0.52^b^	0.33–0.81	10.42^b^	2.33–55.65	1.00^a^	1.00–1.00	1.54^c^	1.23–1.97	1.01^b^	1.00–1.02	259.63^c^	26.58–3311.14
HTN	619 (56.8%)	1.15^b^	1.02–1.29	0.61^c^	0.45–0.80	5.02^c^	2.01–13.44	1.00^a^	1.00–1.00	1.48^c^	1.26–1.74	1.01^b^	1.00–1.01	94.20^c^	22.57–434.69
H.TGs^d^	237 (21.8%)	1.39^b^	1.10–1.81	0.73^a^	0.41–1.21	3.17^a^	0.86–12.79	1.00^a^	1.00–1.01	1.93^c^	1.34–2.88	1.01^b^	1.00–1.02	54.25^b^	4.25–1,188.40
H.CHOL^e^	71 (6.5%)	1.12^a^	0.71–1.66	0.16^b^	0.03–0.64	1.69^a^	0.16–0.30	1.00^a^	1.00–1.01	1.84^b^	1.11–3.29	1.02^b^	1.01–1.04	3,211.41^b^	6.50–9685020.36

*The ORs (95% CI) of inflammatory indicators on the AS risk in four different populations from the training set by univariate logistic regression*.

*Independent variable: leukocyte subtype counts and ratios*.

*Dependent variable: outcome of AS*.

*Population: four subgroups of each pathophysiological characteristic in the training set*.

a *P > 0.05;*

b *P <0.05;*

c *P <0.001;*

d *TGs ≥2.3 mmol/L;*

e *CHOL ≥6.2 mmol/L*.

### Model Evaluation in Training Set and Validation Set

**Figure 3:**
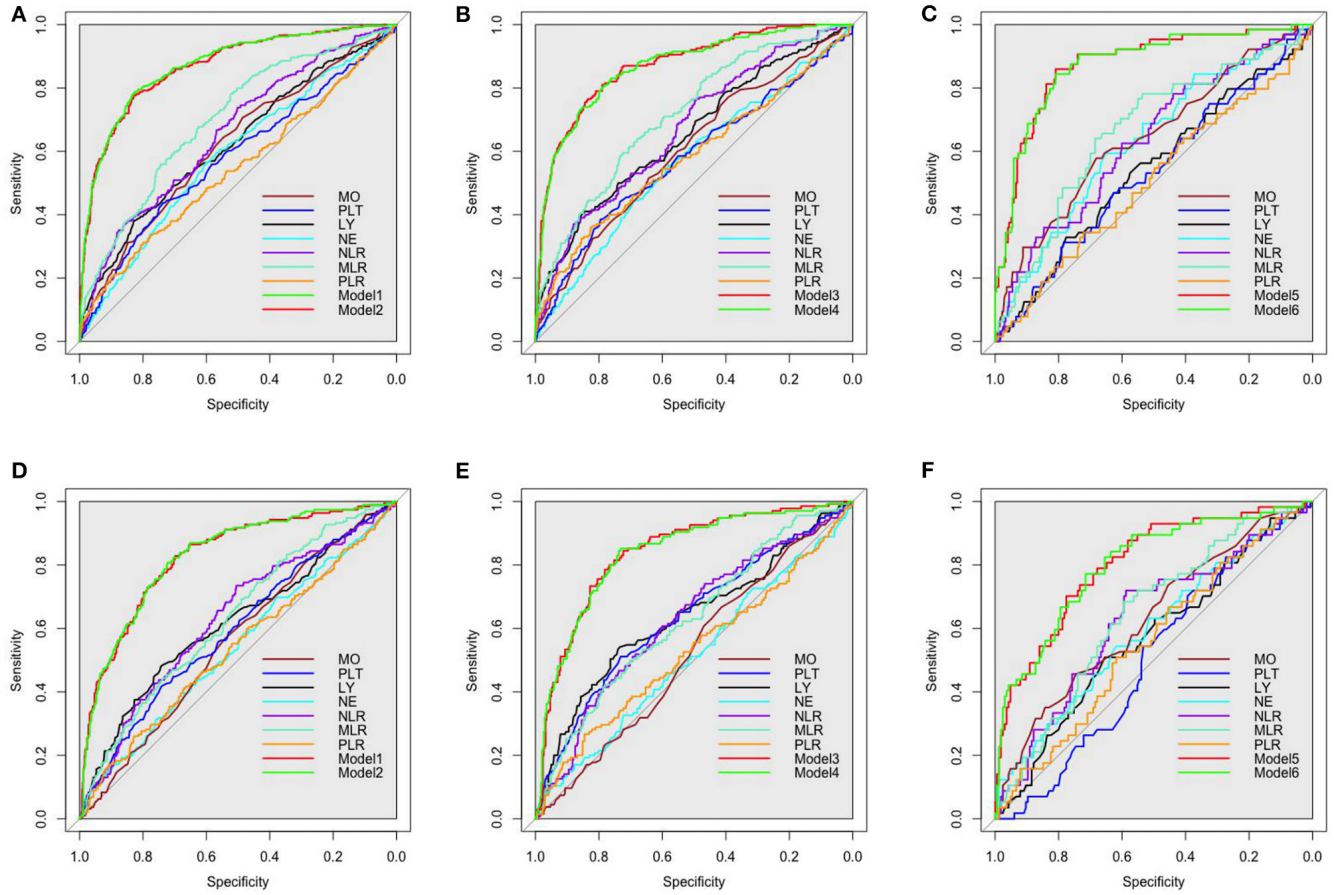
Receiver operating characteristic (ROC) curves of all inflammatory indicators for identifying arterial stiffness in training set. **(A)** The all participants in training set. **(B)** the men in training set. **(C)** The women in training set. **(D)** The all participants in validation set. **(E)** The men in validation set. **(F)** The women in validation set. The models were made by multivariate linear regression analysis on CAVI with backward regression (Table 3 summarizes the specific variables and coefficients included in the models). LY, lymphocyte; NE, neutrophil; MO, monocyte; PLT, platelet; NLR, neutrophil–lymphocyte ratio; MLR, monocyte–lymphocyte ratio; PLR, platelet–lymphocyte ratio.

The ROCs of inflammation cell counts and ratios and the six models (Table 4) in multiple regression of CAVI for diagnosing arterial stiffness in training set and validation set are shown in Figure 3. Among the individual indicators, MLR had the largest area under the ROC curve (AUC), which was 0.63–0.72 in different populations of the training set and validation set. There was no significant difference in the efficacy of the six models in diagnosing arterial stiffness (*p* > 0.05), and their AUCs were all 0.87 in the training set and 0.80–0.84 in the validation set (Table 7).

**Table 7:** ROC curves of inflammatory indicators and models ^a^for identifying arterial stiffness.

	**Training set**					**Validation set**				
	**AUC (95% CI)**	**Specificity**	**Sensitivity**	**Cutoff**	**Youden index**	**AUC (95% CI)**	**Specificity**	**Sensitivity**	**Cutoff**	**Youden index**
LY^b^	0.62 (0.59–0.66)	0.83	0.38	1.39	0.21	0.61 (0.57–0.66)	0.74	0.48	1.49	0.22
NE^b^	0.59 (0.55–0.63)	0.60	0.57	3.37	0.17	0.54 (0.49–0.59)	0.38	0.70	2.80	0.08
MO^b^	0.62 (0.58–0.66)	0.49	0.71	0.34	0.20	0.56 (0.51–0.60)	0.54	0.57	0.37	0.11
PLT^b^	0.58 (0.54–0.62)	0.74	0.44	177.50	0.18	0.59 (0.54–0.64)	0.74	0.42	178.50	0.16
NLR^b^	0.66 (0.62–0.70)	0.50	0.74	1.68	0.24	0.63 (0.58–0.67)	0.51	0.73	1.74	0.24
MLR^b^	0.70 (0.66–0.74)	0.52	0.78	0.19	0.30	0.63 (0.58–0.68)	0.44	0.75	0.18	0.19
PLR^b^	0.54 (0.50–0.59)	0.78	0.33	141.87	0.11	0.54 (0.49–0.58)	0.83	0.26	159.18	0.09
Model 1^b^	0.87 (0.85–0.90)	0.83	0.79	0.25	0.62	0.83 (0.79–0.86)	0.72	0.81	0.24	0.53
Model 2^b^	0.87 (0.84–0.89)	0.83	0.78	0.26	0.61	0.82 (0.79–0.86)	0.68	0.84	0.22	0.52
LY^c^	0.65 (0.61–0.70)	0.85	0.41	1.39	0.26	0.63 (0.58–0.69)	0.74	0.54	1.50	0.28
NE^c^	0.57 (0.52–0.62)	0.69	0.45	3.79	0.14	0.51 (0.45–0.57)	0.95	0.10	5.74	0.05
MO^c^	0.61 (0.56–0.65)	0.39	0.77	0.33	0.16	0.52 (0.47–0.58)	0.41	0.67	0.35	0.08
PLT^c^	0.58 (0.53–0.63)	0.80	0.39	166.50	0.19	0.64 (0.58–0.69)	0.73	0.51	178.50	0.24
NLR^c^	0.67 (0.63–0.71)	0.50	0.77	1.68	0.27	0.63 (0.57–0.68)	0.60	0.61	1.99	0.21
MLR^c^	0.72 (0.67–0.76)	0.73	0.60	0.24	0.33	0.63 (0.57–0.68)	0.78	0.42	0.28	0.20
PLR^c^	0.58 (0.53–0.63)	0.83	0.36	141.31	0.11	0.53 (0.47–0.59)	0.85	0.27	159.06	0.12
Model 3^c^	0.87 (0.84–0.90)	0.84	0.77	0.27	0.61	0.84 (0.80–0.88)	0.72	0.84	0.23	0.56
Model 4^c^	0.87 (0.84–0.90)	0.79	0.81	0.22	0.60	0.84 (0.80–0.88)	0.73	0.85	0.24	0.58
LY^d^	0.54 (0.46–0.62)	0.58	0.53	1.70	0.11	0.57 (0.48–0.65)	0.66	0.51	1.61	0.17
NE^d^	0.63 (0.56–0.71)	0.65	0.59	3.28	0.24	0.59 (0.51–0.68)	0.62	0.54	3.10	0.16
MO^d^	0.64 (0.56–0.72)	0.68	0.58	0.37	0.26	0.63 (0.54–0.71)	0.75	0.46	0.38	0.21
PLT^d^	0.53 (0.45–0.61)	0.78	0.31	180.00	0.09	0.50 (0.42–0.59)	0.27	0.83	252.50	0.10
NLR^d^	0.63 (0.56–0.71)	0.60	0.63	1.79	0.23	0.63 (0.54–0.71)	0.59	0.72	1.73	0.31
MLR^d^	0.67 (0.59–0.74)	0.69	0.64	0.20	0.33	0.64 (0.56–0.72)	0.59	0.68	0.18	0.27
PLR^d^	0.51 (0.42–0.59)	0.72	0.34	102.73	0.06	0.55 (0.47–0.64)	0.62	0.51	136.34	0.13
Model 5^d^	0.87 (0.83–0.92)	0.81	0.86	0.20	0.67	0.80 (0.74–0.87)	0.78	0.70	0.30	0.48
Model 6^d^	0.87 (0.82–0.92)	0.81	0.84	0.22	0.65	0.80 (0.73–0.87)	0.72	0.77	0.26	0.49

*Recognition ability for arterial stiffness of inflammatory indicators and multiple regression models was assessed by ROC curves analysis*.

a *The multivariate linear regression models identifying influencing factors that predict CAVI in training set with backward selection method (in Table 3)*.

b *In all participants*.

c *In men*.

d *In wome*n.

*ROC, receiver operating characteristics; AUC, areas under the ROC curve; BMI, body mass index; LY, lymphocyte; NE, neutrophil; MO, monocyte; PLT, platelet; NLR, neutrophil–lymphocyte ratio; MLR, monocyte–lymphocyte ratio; PLR, platelet–lymphocyte ratio; UA, uric acid; FBG, fasting blood glucose; HTN, hypertension; DM, diabetes*.

## Discussion

Arterial stiffness and atherosclerosis are the main manifestations of vascular aging, and inflammation is considered to be a common pathological feature in this process, which might break the balance between the breakdown and synthesis of elastin (21).

Our research confirms and emphasizes the role of MO, especially the MLR, in the process of arterial stiffness. A large-scale community study in Japan found that there was a significant correlation between the white blood cell and CAVI in men (22). And the independent predictive effect of MO count on IMT and plaque formation of the common carotid artery could be better than interleukin 6, fibrinogen, and white blood cell (15). However, less research has analyzed the association between inflammatory cell count or ratio and arterial elasticity assessed by CAVI. Our study directly described the relationship between count or the ratio of leukocyte subtypes and CAVI and assessed the risk on arterial stiffness of these indicators in men and women. MO performs better than other leukocytes in either the univariate linear regression with CAVI, the logistic regression for the risk of arterial stiffness, or the ROC for indicating arterial stiffness. One standard deviation (0.18 G/L) increase in MO counts leads to an increase in risk of arterial stiffness by 487%, and the AUC is 0.62. In a prospective study by Johnsen et al. (16), an increase in MO count by 1 standard deviation would increase the risk of developing to a higher plaque classification by 18%. All of this is closely related to the core process of monocytes migrating to the blood vessel wall during the development of atherosclerosis, transforming into intimal macrophages, and then generating foam cells by imbibing lipid.

LY constitutes an important part of the body's immune system, and the development of atherosclerosis and increased plaque instability are accompanied by the apoptosis of LY, which play an anti-inflammatory effect against the inflammatory process of atherosclerosis (23, 24). The MLR combines the increase in the risk factor of MO and the decrease in the protective factor of LY, which has a dual risk effect on arterial stiffness. A cutpoint MLR of 0.19 had 70% accuracy for identifying arterial stiffness in all participants. And those cutoffs are 0.24 in men and 0.20 in women. In other researches on the predictive capability of MLR, an optimal cutoff point for predicting carotid artery stenosis in ischemic stroke population is 0.20 (13), and that for predicting long-term major adverse cardiac event in non–ST-elevated myocardial infarction patients is 0.31 (12). The cutoff point calculated in this article for predicting arterial stiffness is at a low level in the current study to predict various clinical events. It may be because arterial stiffness is a common early pathological change to these outcomes. The MLR has been considered to have great value in the diagnosis of malignant tumors (25, 26) and infectious diseases (27, 28). At present, there has been more progress in the cognition of its value in chronic diseases such as cardiovascular disease, cerebrovascular disease, and chronic kidney disease (13, 29). Recognizing the connection between MLR and CAVI helps people understand the mechanism of arterial stiffness from the perspective of inflammation and discover the potential clinical value of MLR in identifying arterial stiffness.

Besides, by subgroup analysis stratified by sex, we found that the intensity of connection between MLR and CAVI is higher in men than in women, as demonstrated in the weights of MLR on CAVI in men was 12.87%, whereas in women, MLR was not retained in the final model. The OR of MLR on arterial stiffness was 28.95 (*p* < 0.001) in men, but it was non-significant in women (*p* > 0.05); the efficacy of MLR in suggesting arterial stiffness in men is better than in women (AUC = 0.72 vs. 0.67). The intimate association between MLR and CAVI in men may confirm the fact that men are more susceptible to ischemic heart disease of large vessels than women (30). Because of the anti-inflammatory effects of estrogen on immune and endothelial cells, the progression of arteriosclerosis in premenopausal women is relatively more sluggish than age-matched men (31). The increase in chronic inflammation during menopause may promote the progression of arterial stiffness (32). The previous studies (33–35) had noted the gender difference in the relationship between lymphocyte count and arterial stiffness. In line with previous studies, we have shown that arterial elasticity itself and risk factors for arterial stiffness differ on gender, which may be the gender differences foundation for the epidemiology, pathogenesis, and manifestations of cardiocerebrovascular disease. That emphasizes the necessity of analyzing the relationship between lymphocyte subtype counts or ratios and CAVI in gender-stratified subgroups.

Our study also has several limitations. In terms of the causal relationship between the observed indicators and the occurrence of arterial stiffness, the following multicenter, prospective cohort study will be more convincing. In addition, considering the significant differences in the progression of arterial stiffness in premenopausal and postmenopausal women, it is necessary to perform a subgroup analysis in women around menopause. Finally, the inflammatory indicators evaluated in our study are limited. Further inclusion of more indicators, such as C-reactive protein, the ratio of monocytes to high-density CHOL, the ratio of C-reactive protein to albumin, etc., may bring about more comprehensive findings.

In conclusion, there is a significant linear relationship between MLR and CAVI, and the relationship between MLR and CAVI is closer in men than in women. MLR is an extremely high-risk factor for arterial stiffness, especially in men. Therefore, MLR may be regarded as a potential indicator for predicting the occurrence of arterial stiffness, and the model combined with general clinical data is of great significance to predicting arterial stiffness.

## Data Availability Statement

The datasets presented in this study can be found in online repositories. The names of the repository/repositories and accession number(s) can be found below: https://doi.org/10.7910/DVN/SGHUV2.

## Ethics Statement

Ethical review and approval was not required for the study on human participants in accordance with the local legislation and institutional requirements. Written informed consent for participation was not required for this study in accordance with the national legislation and the institutional requirements.

## Author Contributions

BQ: conceptualization. RW, YW, YL, LB, LL, and LH: methodology and data collection. YW: data curation, formal analysis, visualization, and writing—original draft. RW: supervision and writing—review & editing. All authors contributed to the article and approved the submitted version.

## Conflict of Interest

The authors declare that the research was conducted in the absence of any commercial or financial relationships that could be construed as a potential conflict of interest.
